# Carrier type inversion in quasi-free standing graphene: studies of local electronic and structural properties

**DOI:** 10.1038/srep10505

**Published:** 2015-06-01

**Authors:** Christos Melios, Vishal Panchal, Cristina E. Giusca, Włodek Strupiński, S. Ravi P. Silva, Olga Kazakova

**Affiliations:** 1 National Physical Laboratory, Teddington, TW11 0LW, United Kingdom; 2 Advanced Technology Institute, University of Surrey, Guildford, Surrey, GU2 7XH, UK; 3 Institute of Electronic Materials Technology, Wólczyńska 133, 01-919 Warsaw, Poland

## Abstract

We investigate the local surface potential and Raman characteristics of as-grown and *ex-situ* hydrogen intercalated quasi-free standing graphene on 4*H*-SiC(0001) grown by chemical vapor deposition. Upon intercalation, transport measurements reveal a change in the carrier type from n- to p-type, accompanied by a more than three-fold increase in carrier mobility, up to *μ*_*h*_ ≈ 4540 cm^2^ V^−1^ s^−1^. On a local scale, Kelvin probe force microscopy provides a complete and detailed map of the surface potential distribution of graphene domains of different thicknesses. Rearrangement of graphene layers upon intercalation to (*n* + 1)LG, where n is the number of graphene layers (LG) before intercalation, is demonstrated. This is accompanied by a significant increase in the work function of the graphene after the H_2_-intercalation, which confirms the change of majority carriers from electrons to holes. Raman spectroscopy and mapping corroborate surface potential studies.

Graphene, a zero band-gap semiconductor consisting of a single layer of sp^2^-bonded carbon atoms, has received significant attention due to its exceptional electronic and mechanical properties^1^. With its π-band displaying linear dispersion around the Dirac point and its electrons behaving like massless Dirac fermions^2^,^3^, graphene is predicted to be a technologically important material in a post-silicon era of analogue high speed electronics^4^,^5^.

A promising route for the production of material suitable for electronics is growth of graphene on SiC, which is a wide band-gap semiconductor. In the present work graphene growth is achieved by the chemical vapor deposition (CVD) method. In this method uniform graphene films are grown by carbon deposition, introduced by a hydrocarbon source, on the Si(0001) side of the SiC wafer^6^. Despite the uniform coverage of the graphene film grown by CVD method, the formation of the interfacial layer (IFL) results in significant electron doping of the graphene, due to charge transfer^7^,^8^ and degradation of mobility due to impurity and phonon scattering^9^. The IFL is a reconstructed carbon layer, topographically similar to graphene, but with a significant number of carbon atoms still covalently bonded to the SiC(0001) surface. This alters the electronic properties of the first graphene layer. Several groups have previously reported decoupling of the IFL from the SiC substrate, using hydrogen intercalation, and its conversion to quasi-free standing graphene (QFSG)^7^,^8^,^10^,^11^. The hydrogen intercalation breaks the C-Si bonds between the IFL and substrate and creates Si-H bonds, as well as passivating dangling bonds. This results in decoupling of the IFL from the substrate and converting it into the new first layer of QFSG^8^. The resulting QFSG exhibits much higher carrier mobility, which is temperature independent and desirable for high-speed electronics^7^. It is worth noting that a fingerprint of the transformation of the pristine graphene to H_2_-intercalated QFSG is the change in majority carriers from electrons to holes^7^,^8^.

While several groups have investigated the structural properties and electronic band structure of H_2_-intercalated graphene^7^,^8^,^10^,^12^, there are currently no layer-specific studies demonstrating the changes in the local electronic properties (e.g. surface potential or work function) after intercalation of graphene. In this paper, we present the effects of H_2_-intercalation on the local electronic and structural properties of the QFSG. The verification of number of graphene layers was achieved using Raman spectroscopy and mapping, whereas a detailed image of the surface potential of the layer structure was constructed using frequency-modulated Kelvin probe force microscopy (FM-KPFM)^31^. The study of the high-resolution surface potential maps with the aid of Raman spectroscopy provided direct evidence of consequent increase in the number of graphene layers upon intercalation (i.e. (*n* + 1)LG, where *n* is the number of graphene layers (LG) before intercalation). This is accompanied by a considerable increase of work function upon intercalation, which is evidence for the change of the carrier type from electrons to holes with the Fermi level straddling either side of the Dirac point as a function of H_2_-intercalation.

## Results

### As-grown graphene sample

Based on the van der Pauw measurements on the as-grown sample, the carrier concentration and electron mobility were determined as *n*_*e*_ ≈ 1.8 × 10^12^ cm^−2^ and *μ*_*e*_ ≈ 1370 cm^2^ V^−1^ s^−1^, respectively.

**Figure 1:**
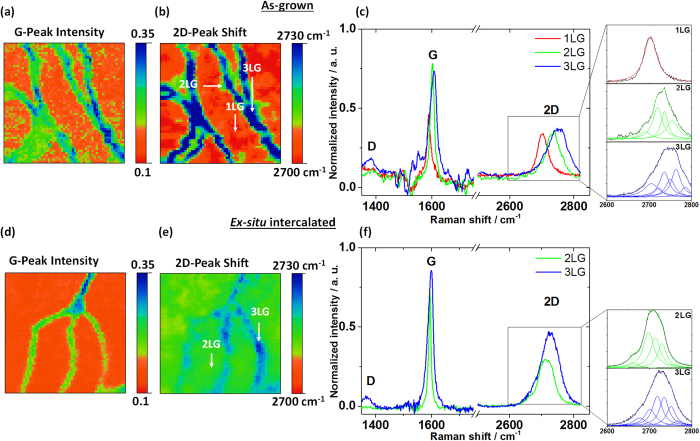
Raman maps and spectra of as-grown and H_2_-intercalated graphene. Raman maps (10×10) μm^2^ of the G peak intensity (**a** and **d**) and 2D peak shift (**b** and **e**) for the as-grown (**a** and **b**) and intercalated (**d** and **e**) samples. Raman spectra taken on the terrace and edges showing: (**c**) for as-grown sample; 1LG, 2LG and 3LG are depicted with red, green and blue lines, respectively; (**f**) for intercalated sample; 2LG and 3LG are depicted with green and blue lines, respectively. The insets in (**c**) and (**f**) show the selected 2D peaks fitted with Lorentzians.

To investigate the layer structure of the as-grown graphene sample, Raman spectroscopy and mapping were employed (Figs. 1a–c). G peak intensity and 2D peak shift Raman maps presented in Fig. 1a,b, respectively, clearly demonstrate two main features: the terraces and terrace edges, covered with graphene of different thicknesses. For additional Raman maps, including intensity, shift and full-width-at-half-maximum (FWHM) of G and 2D peaks, see Supplementary Fig. S1. Three individual spectra taken at the terraces and edges are plotted in Fig. 1c. A summary of the Raman peak analysis is presented in Table 1. The red spectrum in Fig. 1c was collected on the terrace of the graphene sample. The top inset in Fig. 1c shows the 2D peak fitted with a single Lorentzian. The single Lorentzian fitting and the narrow FWHM of 35 cm^–1^ 14, indicate that the areas plotted in red on the Raman map (Fig. 1b) are indeed 1LG. This method was repeated for the green areas on the terrace edges where the G peak exhibits significant increase in intensity (Fig. 1a,c) and the 2D peak is broader than that of 1LG (FWHM = 62 cm^–1^). Moreover, the 2D peak at the terrace edge is blue-shifted (by taking the position of the maximum of the overall fit) towards higher wavenumbers by ~33 cm^–1^ compared to 1LG (Fig. 1b,c). This peak shows the typical line shape of *AB* stacked 2LG and can be fitted with four Lorentzians^15^,^16^. While the G peak intensity can be influenced by the twist angle between 2 graphene layers that are not *AB* stacked^17^, the 2D peak shift and line shape gives a better indication of the number of layers in this particular case. A representative spectrum collected from the blue area of the terrace edge is plotted in blue in Fig. 1c. This blue-shifted 2D peak (~48 cm^–1^ and ~15 cm^–1^ compared to the 1LG and 2LG 2D peak, respectively) is much broader (FWHM = 75 cm^–1^) than that of 1LG and 2LG, possibly indicating the presence of 3LG. There are some reports in the literature showing that fitting the line shape of graphene with 6 Lorentzian components is an indication of 3LG^18^. However, it is important to point out that, while our fit of 1 and 2LG with one and four Lorentzians, respectively, clearly shows the expected line shape of 1 and 2LG, the fitting of 3LG with 6 Lorentzians is not entirely justified given the spatial resolution of the system. In this case, the Raman signal contains contributions from both 2 and 3LG. The same could be true for 2LG, as the signal might potentially contain contributions from 1LG, however the area of 2LG where the representative spectrum was taken (Fig. 1c) is larger than the spatial resolution of our system.

Some small variations of the 2D peak shift (~6 cm^−1^) within the terrace are visible in Fig. 1b, making non-uniform areas of ~1 μm in size. Additionally, deviations of the G peak shift (~4 cm^−1^) have been measured and presented in Supplementary Information Fig. S1. It has been shown that in graphene on SiC the presence of residual strain in the carbon lattice can result in variations in the 2D peak shift^19^. Furthermore, these variations may also be related to charge inhomogeneities^20^,^21^. Since the 2D peak in graphene is directly related to the Fermi energy, the 2D peak shift can be additionally influenced by doping. In particular, because of the linear dispersion relation, 1LG is far more sensitive to doping than thicker layers, where the dispersion relation is parabolic. While many groups^15^,^20^,^21^ use the position of G and 2D peaks as a powerful technique to measure the carrier concentration of exfoliated graphene on SiO_2_, using these studies as a reference to determine the doping and charge inhomogeneities in CVD graphene on SiC would be inaccurate, since the interactions between graphene and supporting substrate are different. Thus, the combination of strain and charge carrier coupling may be the origin of the fluctuations of 2D and G peak positions on the terraces^19^.

**Figure 2:**
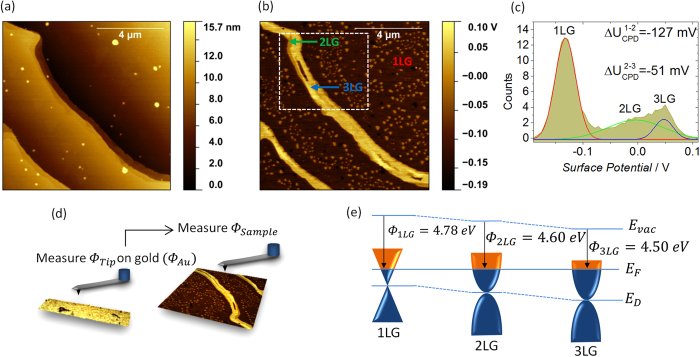
Topography, surface potential and work function measurements of the as-grown graphene. (**a**) Topography and (**b**) surface potential maps of the as-grown sample, showing terraces covered by continuous 1LG with individual 2LG islands as well as elongated 2LG and 3LG domains at the edges. (**c**) The surface potential histogram of the framed area in (**b**) fitted with three contrast levels corresponding to 1LG, 2LG and 3LG. (**d**) Schematic representation of the quantitative work function measurements, by initially calibrating the tip work function against a known gold sample. (**e**) Schematic representation of energy band diagrams for 1LG, 2LG and 3LG.

To further evaluate and resolve the different graphene layers in the as-grown sample, FM-KPFM was used to produce the topography and surface potential maps, shown in Fig. 2a,b, respectively. Figure 2a reveals terrace are ~4 μm wide and ~5 nm high. The representative 10 × 10 μm^2^ map of the surface potential reveals SiC terraces covered by a continuous layer of 1LG (Fig. 2b). 2LG covers a small portion of the terrace edges (see a narrow band in top left corner of Fig. 2b), while most of them are covered with 3LG (exhibiting the brightest contrast). In addition to these main features, the terraces are additionally decorated by 2LG islands of ~500 nm in size, as identified from their contrast. Both substrate preparation and CVD growth conditions may result in formation of these 2LG islands. For further assessment of the different contrast levels, the histogram in Fig. 2c was used.

For the as-grown sample, we report the difference in the surface potential (Δ*U*_*CPD*_) between 1LG and 2LG to be 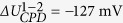 and between 2LG and 3LG - 
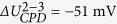
. When tip-biased is used, the increase in the surface potential and subsequently the brightness of FM-KPFM images, with the increasing number of layers is characteristic for n-type doping. For practical applications, it is often important to define the work function of the material. To quantitatively measure the work function of each graphene layer, the work function of the scanning probe microscopy (SPM) tip was calibrated to be *Φ*_*Tip*_ = 4.52 ± 0.05 eV (see Experimental section and Fig. 2d). The schematic energy band diagrams of 1, 2 and 3LG are displayed in Fig. 2e. The corresponding graphene work functions were measured to be *Φ*_*1LG*_ = 4.78 ± 0.03 eV, *Φ*_*2LG*_ = 4.60 ± 0.05 eV and *Φ*_*3LG*_ = 4.50 ± 0.08 eV, respectively (for details regarding tip calibration, see experimental section for details). The decrease in work function (increase of electron carrier concentration) with increasing number of layers confirms the n-type character of the as-grown sample. Having correlated the Raman characteristics with the surface potential maps, it is then possible to conclude that the SiC terraces are indeed covered with 1LG, whereas thicker graphene (2–3LG) grows at terrace edges, resulting in lower local work function.

### *Ex-situ* intercalated graphene sample

The *ex-situ* intercalated sample (i.e. H_2_-intercalation of the as-grown sample described above) was measured using Hall effect in the van der Pauw geometry, where the hole carrier concentration and mobility are *n*_*h*_≈1.5 × 10^13^ cm^−2^ and *μ*_*h*_ ≈ 4540 cm^2^ V^−1^ s^−1^ (i.e. more than three times greater than the as-grown sample), respectively. The transformation of the graphene from an electron to hole-doped material is a fingerprint for the successful intercalation of the sample.

Figures 1d–f show the G peak intensity and 2D peak shift maps of the intercalated sample (for additional Raman mapping, see Supplementary Information, Fig. S2). Similar to the Raman analysis of the as-grown sample, individual representative spectra taken at the terraces and edges are plotted in Fig. 1f and a summary of the analysis is presented in Table 1. In Fig. 1e, the 2D peak of the spectrum taken on the terraces (depicted with green) is significantly blue-shifted compared to the as-grown sample (15 cm^–1^). In addition, the peak is broader with a FWHM of 58 cm^–1^ (top inset of Fig. 1f). The line shape as well as the blue-shift of the 2D peak is a clear indication of *AB* stacked 2LG covering the terraces, which is in agreement with previous reports on intercalated graphene on 6*H*-SiC(0001)^15^,^22^. Analysis of the terrace edges (depicted with blue) demonstrates that the 2D peak has FWHM of ~71 cm^–1^ and can be fitted with six Lorentzians^18^. This further confirms the increased thickness of graphene at the edges, implying that it is now 3LG, or a mixture of 2 and 3LG, given the spatial resolution of the Raman system. It is important to note that 4LG, which was observed in FM-KPFM was not resolved by Raman due to limitations in spatial resolution. By demonstrating that the terraces are covered with 2LG upon H_2_-intercalation, we prove that the IFL, which was under the 1LG in the case of the as-grown sample, is now transformed into the new first graphene layer. This results in the general rearrangement of the graphene layers to (*n* + 1)LG, where *n* is the number of layers before intercalation.

After intercalation, some inhomogeneity of the 2D peak shift map on the terraces (i.e. 2LG) is still observed (Fig. 1e). Compared with the as-grown sample, after intercalation the shifts of the 2D peak position are limited to ~2 cm^−1^ (~1 cm^−1^ for the G peak, see Supplementary Information, Fig. S2). This indirectly supports the quasi-free standing nature of the H_2_-intercalated graphene.

**Figure 3:**
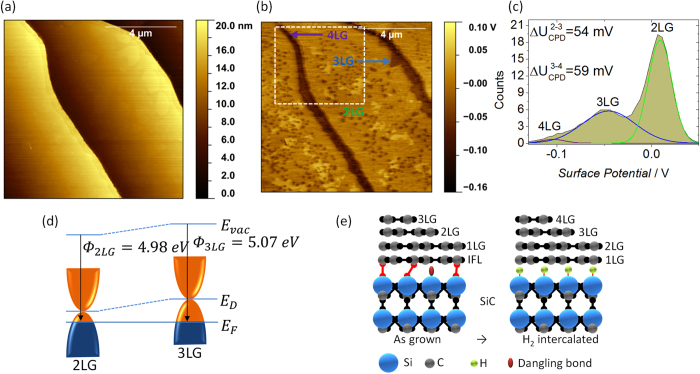
Topography, surface potential and work function measurements of the H_2_-intercalated graphene. (**a**) Topography and (**b**) surface potential map of the *ex-situ* intercalated graphene sample, showing terraces covered with continuous 2LG, 3LG islands and elongated 3LG and 4LG at the terrace edges. (**c**) Surface potential histogram of the framed area in (**b**) fitted with three components, corresponding to 2LG, 3LG and 4LG. (**d**) Schematic representation of energy band diagrams for 2LG and 3LG. (**e**) Schematic representation of the transformation of the as-grown graphene layer structure to quasi-free standing graphene.

Having established the successful transformation of the as-grown graphene to QFSG (it should be noted that the sample maintains its excellent morphology, as presented in the topography map in Fig. 3a), we further consider changes in the surface potential map of the intercalated sample to observe the effect of H_2_-intercalation on the local electronic properties of QFSG. The map of the surface potential and the relevant histogram (Fig. 3b,c) show substantial changes as compared to the as-grown sample. Taking into consideration the three contrast levels and correspondingly correlating the three different regions with the Raman maps presented in Fig. 1d,e, we deduce that the terraces are now completely covered with continuous 2LG and the previously observed 2LG and 3LG features (islands and terrace edges) are now transformed into 3LG and 4LG, respectively. The p-type character of the intercalated sample (hole conductivity) results in the decrease in the surface potential with increasing number of graphene layers. This is attributed to the fact that the *U*_*CPD*_ between the tip and the sample gets more negative with the increasing number of layers (Fig. 3c), which can be inferred by comparing the schematic band diagrams of the as-grown and intercalated graphene in Figs. 2e,3d. Upon intercalation, the surface potential difference between 2LG and 3LG is 
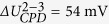
, whereas between 3LG and 4LG is 
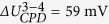
. In addition to the features described above (terrace edges and islands), some bright patches are observed in the middle of the terraces, which show a surface potential difference of ~36 mV with respect to 2LG and results in relatively low contrast difference. These features are of an approximately the same size as inhomogeneities seen in the maps of the 2D peak shift, as seen in Fig. 1e. By analyzing the topography, these patches are elevated by ~ 200 pm with respect to the 2LG. It can be speculated that these features might be due to hydrocarbon species^23^ or hydrogen atoms^8^ trapped underneath the graphene layers, which slightly elevate graphene. The schematic diagram of the energy band structure for 2LG and 3LG of intercalated graphene is shown in Fig. 3d. The work functions for 2LG and 3LG were calculated as *Φ*_*2LG*_ = 4.98 ± 0.03 eV and *Φ*_*3LG*_ = 5.07 ± 0.04 eV, respectively. It should be noted that measurements on the as-grown and intercalated samples were performed using different SPM tips. In the latter case, the calibrated work function of the tip is *Φ*_*Tip*_ = 4.88 ± 0.01 eV. The significant increase in work function as compared to the as-grown sample suggests that the Fermi energy crosses the charge neutrality point, thus providing independent proof that conductivity changes from n- to p-type upon intercalation.

With the annealing of the sample in hydrogen environment at temperatures around 1100 °C, the H_2_ molecule will enter the graphene stack from the terrace edges and defect sites, where it’s position is more energetically favorable^24^. The intercalated hydrogen molecule will then dissociate into H atoms to form Si-H bonds, which will decouple and lift the IFL from the SiC substrate^8^,^24^,^25^ and convert it to 1LG (Fig. 3e). As discussed previously, the intercalation of graphene will transform the as-grown graphene from electron– to hole-doped. The hole-doping of the QFSG can be explained by the spontaneous polarization of the substrate, as for example, shown in Ref.^11^,^26^. In addition to that, by decoupling the IFL, the charge transfer from the SiC is reduced and only environmental p-doping affects the sample. One of the major improvements triggered by the H_2_-intercalation is the significant increase in mobility of the graphene layer. This is typically attributed to the decoupling of the graphene layers from the substrate, where phonon scattering is now suppressed as the main mechanism for the mobility degradation in the case of the as-grown graphene^7^,^27^. Other mechanisms such as the transformation of graphene to graphane (A form of hydrogenated graphene with sp^3^ bonded carbon-hydrogen bonds) has also been examined experimentally and theoretically^13^. In the case of QFSG, the one possible mechanism for mobility degradation is Coulomb scattering from charged impurities^9^,^27^.

## Discussion

We investigated the effects of *ex-situ* H_2_-intercalation of CVD grown graphene on 4*H*-SiC(0001) using bulk transport, local surface potential and Raman spectroscopy and mapping. Transport measurements on the as-grown sample demonstrated n-type doping with *μ*_*e*_ ≈ 1370 cm^2^ V^−1^ s^−1^. Following the *ex-situ* intercalation, the graphene conductivity switched to p-type, which along with a significant increase in mobility, *μ*_*h*_ ≈ 4540 cm^2^ V^−1^ s^−1^, is indicative of successful intercalation. The FM-KPFM measurements of the as-grown sample revealed that SiC terraces were covered by predominantly 1LG and additionally decorated with 2LG islands and 2LG/3LG edges. The work function measurements also indicated an increase in the electron concentration (i.e. decrease in work function) as the number of layers increased. On the contrary, the H_2_-intercalated sample exhibited increase of the work function as the number of graphene layers increased. This is the ultimate proof that the Fermi energy crossed the charge neutrality point from n- to p-type upon intercalation. In addition, for the first time a high-resolution image of the surface potential of intercalated graphene was constructed, which provided a detailed understanding of the layer structure and its transformation upon decoupling from the substrate. The Raman studies proved that upon intercalation the 1LG has been transformed into 2LG and, in general, the as-grown layers (*n*) have been transformed into (*n* + 1)LG, as followed by conversion of the IFL into 1LG.

Thus, using local, layer-resolving techniques, we demonstrate successful transformation of graphene covalently bound to the substrate into QFSG with superior electronic properties. QFSG is one of the preferable candidates for high-speed electronics, as the decoupling of the IFL from the SiC substrate increases the mobility dramatically, while maintaining the excellent intrinsic electronic and topographic structure.

## Methods

### Sample growth and H_2_-intercalation

For this study, graphene samples were grown by CVD method at 1600 °C under an argon laminar flow in an Aixtron VP508 hot-wall reactor. Semi-insulating on-axis oriented 4*H*-SiC (0001) substrates (Cree) 10 × 10 mm^2^ size were cut out from 4” wafer and etched in hydrogen at 1600 °C prior to the epitaxy process. Graphene growth was controlled by Ar pressure, Ar linear flow velocity and reactor temperature. The process relies critically on the creation of dynamic flow conditions in the reactor, which control Si sublimation rate and enable the mass transport of hydrocarbon to the SiC substrate. Tuning the value of the Reynolds number enables formation of an Ar boundary layer, which is thick enough to prevent Si sublimation and allowing diffusion of hydrocarbon to the SiC surface, followed by CVD growth of graphene on the SiC surface. The *ex-situ* intercalation of hydrogen on the same sample was achieved by annealing the sample in molecular hydrogen at temperature of 1100–1200 °C and reactor pressure of 900 mbar. Cooling down in H_2_ atmosphere keeps hydrogen atoms trapped between graphene and substrate. Prior to unloading the sample, the process gas was changed back to argon^6^,^8^.

### Measurements

The mobility and carrier concentration of the as-grown and *ex-situ* hydrogen intercalated samples were characterized using Hall effect measurements in the van der Pauw geometry in ambient conditions.

Raman maps of 10 × 10 μm^2^ were obtained using a Horiba Jobin-Yvon HR800 system in order to investigate the structure of graphene samples. The 532 nm wavelength laser (5.9 mW power) was focused through a 100 × objective lens onto the graphene sample. The spectral resolution was 1.59 cm^–1^. The Raman spectra were initially obtained for a reference SiC substrate, which is then used to subtract the substrate related signal, allowing effective separation of the Raman peaks originating only from the graphene. The Raman maps were constructed by mapping G and 2D peak intensity, shift and FWHM of 3025 individual spectra with XY resolution of 0.2 μm. The G peak (~1582 cm^–1^) originates from the first order scattering process due to the double degenerate phonon mode vibrations at the center of Brillouin zone^15^,^28^,^29^. The 2D peak (~2700 cm^–1^) originates from the double resonance scattering process near the K point. The 2D peak exhibits a dispersive behavior. A characteristic feature of increasing number of graphene layers on SiC is the blue shift of the 2D peak^15^. Furthermore, the 2D peak of 1LG can be fitted with a single Lorentzian, whereas for 2LG and 3LG - with four (indicative of *AB* stacking) and six Lorentzians, respectively^28^,^30^, where some limitations of the fitting process are discussed in the text.

Bruker Dimension Icon scanning probe microscope was employed to investigate the surface potential of the graphene samples in ambient conditions. Despite the high resolution of an atomic force microscope, traditional topography maps cannot be reliably used to identify the number of layers in graphene grown on SiC^30^,^31^,^32^,^33^. For this study, doped Si PFQNE-AL tips (Bruker) with a spring constant *k* ≈ 0.4–1.2 N m^−1^ were used in single-pass tapping mode. In FM-KPFM, the cantilever is oscillating at its mechanical resonant frequency *f*_*0*_ ≈ 300 kHz, in addition to a much lower AC frequency voltage, f_mod_ ≈ 5 kHz and V_mod_ ≈2-5 V, to induce a frequency shift (*f*_*0*_ ± f_mod_). The *f*_*0*_ ± f_mod_ side lobes are monitored by a PID feedback loop, which applies a compensation DC voltage to minimize the side lobes, thus acquiring the surface potential map. Since FM-KPFM detects the force gradient using the frequency shift, it can achieve spatial resolution of <20 nm, which is limited only by the tip apex diameter^32^,^34^,^35^. Thus, FM-KPFM allows determining the surface potential [i.e. contact potential difference (*U*_*CPD*_)] with great accuracy for the number of graphene layers. To further investigate the surface potential of graphene samples, the work function of the PFQNE-AL tip (*Φ*_*TIP*_) was calibrated against a gold reference sample using the approximation *Φ*_*Tip*_ ≈ *Φ*_*Au*_ + e*U*_*CPD*_ (Fig. 2d), where the work function of gold *Φ*_*Au*_ = 4.99 ± 0.003 eV was measured using ultra-violet photoelectron spectroscopy^31^. Following the tip work function calibration, small areas (~2-5 μm) of the graphene samples were scanned and by using *Φ*_*Sample*_ ≈ *Φ*_*Tip*_ -*U*_*CPD*_, a work function was determined for each graphene layer. It is important to stress that the surface potential maps and work function measurements were performed on different days; therefore, variations in the relative humidity of the ambient air may lead to changes to the surface doping, justifying the discrepancy.

## Additional Information

**How to cite this article**: Melios, C. *et al.* Carrier type inversion in quasi-free standing graphene: studies of local electronic and structural properties. *Sci. Rep.* **5**, 10505; doi: 10.1038/srep10505 (2015).

## Supplementary Material

Supporting Information

## Figures and Tables

**Table 1:** Summary of Raman characteristics of G and 2D peaks in as-grown and H_2_-intercalated graphene samples.

		**2D position [cm**^**−1**^]	**2D FWHM [cm**^**−1**^]	**Number of Lorentzian fits**	**2D Shift against 1LG [cm**^**−1**^]	**G position [cm**^**−1**^]	**G FWHM [cm**^**−1**^]	**Number of layers**
As-grown	Terrace	2702	35	1	−	1589	14	1
	Edge	2735	62	4	33	1597	16	2
	Edge	2750	75	6	48	1600	34	3
Intercalated	Terrace	2717	58	4	15	1594	10	2
	Edge	2730	71	6	28	1599	23	3
