# Supplementary material for: Carrier type inversion in quasi-free standing graphene: studies of local electronic and structural properties
Source: Sci Rep. 2015 Jun 1;5:10505. doi: 10.1038/srep10505 (PMC4450755; doi:10.1038/srep10505)
Supplement: Supporting Information [file srep10505-s1.pdf]

Carrier type inversion in quasi-free standing graphene: studies of local electronic and structural properties

Christos Melios[1,2], Vishal Panchal[1], Cristina E. Giusca[1], Włodek Strupiński[3], S. Ravi P. Silva[2] and Olga Kazakova[1]*

[1]National Physical Laboratory, Teddington, TW11 0LW, United Kingdom, [2]Advanced Technology Institute, University of Surrey, Guildford, Surrey, GU2 7XH, UK, [3]Institute of Electronic Materials Technology, Wólczyńska 133, 01-919 Warsaw, Poland

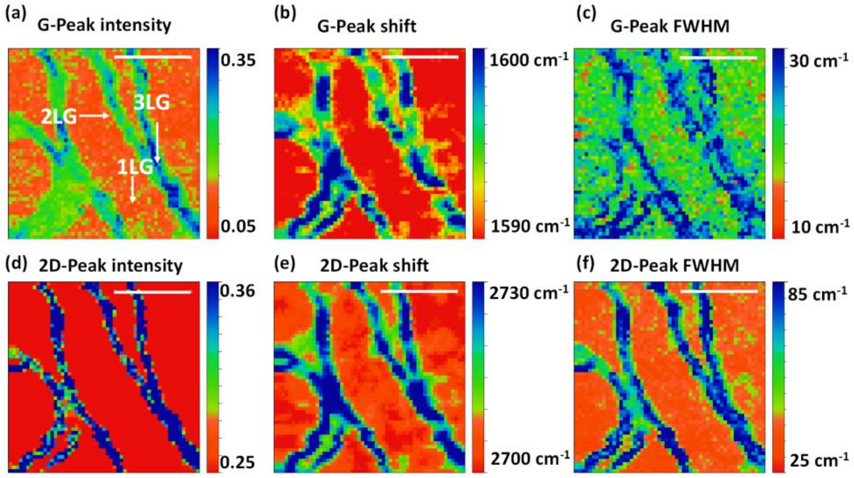

Figure S1 | Raman maps of as-grown graphene. Raman maps (10×10) μm^2 of (a) G peak intensity, (b) G peak shift, (c) G peak FWHM, (d) 2D peak intensity, (e) 2D peak shift and (f) 2D peak FWHM. Scale bars are 4 μm.

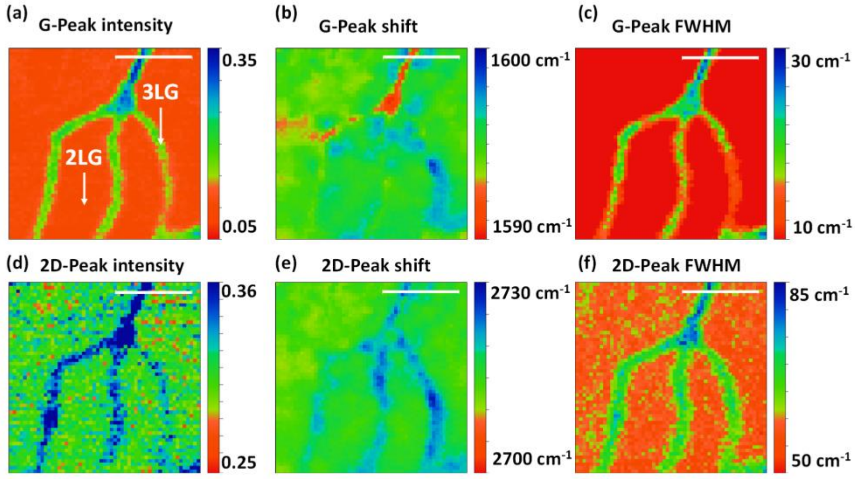

Figure S2 | Raman maps of *ex-situ* intercalated graphene. Raman maps (10×10) μm^2 of (a) G peak intensity, (b) G peak shift, (c) G peak FWHM, (d) 2D peak intensity, (e) 2D peak shift and (f) 2D peak FWHM. Scale bars are 4 μm.
